# Characterization of the Exo-Metabolome of the Emergent Phytopathogen *Fusarium kuroshium* sp. nov., a Causal Agent of *Fusarium* Dieback

**DOI:** 10.3390/toxins13040268

**Published:** 2021-04-09

**Authors:** Angélica Gutiérrez-Sánchez, Javier Plasencia, Juan L. Monribot-Villanueva, José B. Rodríguez-Haas, Jose Abel López-Buenfil, Clemente J. García-Ávila, Eliel Ruiz-May, Diana Sánchez-Rangel, José A. Guerrero-Analco

**Affiliations:** 1 Laboratorios de Fitopatología y Biología Molecular, Red de Estudios Moleculares Avanzados, Clúster BioMimic^®^, Instituto de Ecología, A. C. Xalapa, Veracruz 91073, Mexico; angelica.gutierrez@posgrado.ecologia.edu.mx (A.G.-S.); benjamin.rodriguez@inecol.mx (J.B.R.-H.); 2 Laboratorio de Química de Productos Naturales, Red de Estudios Moleculares Avanzados, Clúster BioMimic^®^, Instiuto de Ecología, A. C. Xalapa, Veracruz 91073, Mexico; juan.monribot@inecol.mx; 3 Departamento de Bioquímica, Facultad de Química, Universidad Nacional Autónoma de México, Ciudad de México 04510, Mexico; javierp@unam.mx; 4 Colegio de Postgraduados, Carretera México-Texcoco Km 36.5 Texcoco, Estado de México 56101, Mexico; abel.lopez@colpos.mx; 5 Centro Nacional de Referencia Fitosanitaria del Servicio Nacional de Sanidad, Inocuidad y Calidad Agroalimentaria, Tecámac, Estado de México 55740, Mexico; clemente.garcia@senasica.gob.mx; 6 Laboratorio de Proteómica, Red de Estudios Moleculares Avanzados, Clúster BioMimic^®^, Instituto de Ecología, A. C. Xalapa, Veracruz 91073, Mexico; eliel.ruiz@inecol.mx; 7 Cátedra CONACyT en la Red de Estudios Moleculares Avanzados del Instituto de Ecología, A. C. (INECOL), Carretera antigua a Coatepec 351, El Haya, Xalapa, Veracruz 91070, Mexico

**Keywords:** exo-metabolome, fusaric acid, *Fusarium* dieback, *Fusarium kuroshium*, fungal metabolomics, *Persea americana* (avocado), phytotoxicity

## Abstract

*Fusarium kuroshium* is the fungal symbiont associated with the ambrosia beetle *Euwallacea kuroshio*, a plague complex that attacks avocado, among other hosts, causing a disease named *Fusarium* dieback (FD). However, the contribution of *F. kuroshium* to the establishment of this disease remains unknown. To advance the understanding of *F. kuroshium* pathogenicity, we profiled its exo-metabolome through metabolomics tools based on accurate mass spectrometry. We found that *F. kuroshium* can produce several key metabolites with phytotoxicity properties and other compounds with unknown functions. Among the metabolites identified in the fungal exo-metabolome, fusaric acid (FA) was further studied due to its phytotoxicity and relevance as a virulence factor. We tested both FA and organic extracts from *F. kuroshium* at various dilutions in avocado foliar tissue and found that they caused necrosis and chlorosis, resembling symptoms similar to those observed in FD. This study reports for first-time insights regarding *F. kuroshium* associated with its virulence, which could lead to the potential development of diagnostic and management tools of FD disease and provides a basis for understanding the interaction of *F. kuroshium* with its host plants.

## 1. Introduction

Ambrosia fungi belong to the Ascomycota phylum that presents a mutualism with ambrosia beetles (Coleoptera: Curculionidae: Scolytinae and Platypodinae) [1,2]. Both organisms benefit from this association since the fungus gains dispersal, nutrition, physical and antimicrobial defense, while the insect acquires both a source of nutrition and protection against microbial pathogens [3,4]. Ambrosia beetles have a fungal spore-carrying organ called the mycangium, which ensures the vertical transmission of the mutualist and guarantees fungal dispersion to new host trees from different families, such as Fabaceae Lauraceae, Platanaceae, Euphorbiaceae, among others [3,5]. Attention has recently turned to the ambrosia fungi; specifically, those that have been identified as causal agents of emerging diseases, such as *Fusarium kuroshium*, the symbiont of the invasive Asian ambrosia beetle Kuroshio shot hole borer (KSHB) formally named *Euwallacea kuroshio* Gomez and Hulcr [6,7]. This ambrosia complex is the causal agent of *Fusarium* dieback (FD), a disease that severely threatens natural forests, landscape trees and avocado orchards since 2012 when it was identified in San Diego County, USA [8]. In Mexico, the first FD case was reported in 2016 in Baja California [9], and it is currently classified as a quarantine pest [10]. Eskalen and colleagues [5] reported that *F. kuroshium* is not the only fungus associated with the beetle KSHB since *Graphium kuroshium* was identified as part of this complex and confirmed by Koch’s postulates that both fungi are capable of causing disease in healthy young avocado plants. The host range of this complex features over 250 plant species, including either economically important crops or forestry species [11].

As a result of the growing threat imposed by this complex disease, efforts have been focused on the development of molecular tools to rapidly identify *F. kuroshium* and distinguish it from other fungi [12]. However, to achieve this, more information about the mechanisms of pathogenicity of this species is required. In this sense, it is assumed that the fungus is vectored by the insect, thus gaining free access to the host’s xylem [13], and FD is the consequence of fungal mass accumulation on stressed trees, among other possibilities [4]. Since *Fusarium* comprises many species that cause human and plant diseases worldwide [14], an important question would be to determine its contribution to pathogenesis. *Fusarium* species produce a wide range of hydrolytic enzymes that facilitate plant host tissue colonization and a diverse assemblage of toxins, such as fumonisins, fusaric acid (FA), trichothecenes and moniliformin, with phytotoxic properties [15]. The mechanism of action and precise role in the pathogenesis of these mycotoxins are not fully understood [15]. Genomics and transcriptomics approaches, undertaken to understand the pathogenicity of *F. kuroshium*, revealed that the species could potentially produce enzymes responsible for the biosynthesis of FA and other metabolites, as well as synthesizing proteases and ABC transporters [16,17]. To gain the first insights into the role of *F. kuroshium* in this complex disease, we, therefore, performed an exo-metabolomics profile analysis of *F. kuroshium* to identify molecules with phytotoxic properties and conducted in vitro assays in avocado leaves.

## 2. Results

### 2.1. Analysis of F. kuroshium Exo-Metabolome

**Figure 1:**
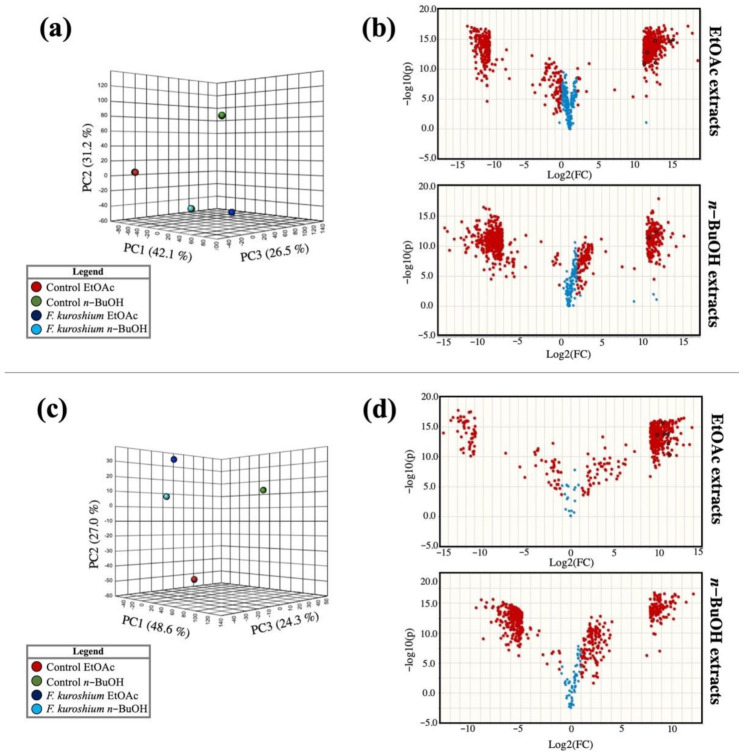
Principal component analysis (PCA) (x: log10 (FC), y: −log10 (*p*-value)) of EtOAc and *n*-BuOH extracts in electrospray ionization ESI^+^ (**a**) and ESI^−^ (**b**) mode. Volcano plots of media inoculated with *F. kuroshium* compared to the control analyzed by UHPLC-MS-QTOF in ESI^+^ (**c**) and ESI^−^ (**d**) mode.

After the *F. kuroshium* culture and analysis by LC–MS, we obtained the mass spectrometric (MS) data of the fungal metabolome and non-inoculated media. Figure 1 shows the principal component analysis (PCA) of the samples analyzed by electrospray ionization (ESI). Figure 1a shows the PCA of the samples analyzed in positive mode (ESI^+^) with explained variances of 42.1, 31.2 and 26.5% for PC1, PC2 and PC3, respectively, indicating a clear separation between the media inoculated with *F. kuroshium* and the control culture media and revealing the ability of *F. kuroshium* to secrete metabolites to the extracellular medium. However, if we only compare the ethyl acetate (EtOAc) extract versus the *n*-butanol (*n*-BuOH) extract of media inoculated with *F. kuroshium*, we note that they are closer in the graph, implying that the solvents used may extract similar or chemically related compounds. Similar behavior occurred with the samples analyzed by electrospray ionization in negative mode (ESI^−^) (Figure 1c) with explained variances of 48.6, 27.0 and 24.3% for PC1, PC2 and PC3, respectively.

Fold change analyses were performed and represented as volcano plots (Figure 1b,d) to compare each extract (EtOAc and *n*-BuOH) with its own control (non-inoculated media). This analysis allowed detecting a total of 1252 mass features secreted by *F. kuroshium* (Supplementary materials, File S1). By ESI^+^ (Figure 1b), 461 features were obtained in the EtOAc extract and 244 for *n*-BuOH. By ESI^−^ (Figure 1d), 353 features were detected in EtOAc and 194 in *n*-BuOH. Due to the high number of *m/z* values detected, the 50 most statistically significant mass features of each extract were prioritized according to their annotations in public databases.

After an extensive search of the selected 200 *m/z* values, eleven molecules were identified with an acceptable level of accuracy; nine in EtOAc, one in *n*-BuOH and one in both extracts (Table 1). According to the Metabolomics Standards Initiative [18], six of these molecules were putatively identified by the coincidence of their MS fragmentation pattern and molecular ions (level 2), five were putatively identified based only on the *m/z* values of their molecular ions (level 3), and more than 1000 *m/z* ratios remain unidentified (level 4). Altogether, this analysis serves as a guide with which to identify molecules produced by *F. kuroshium* that could be isolated and potentially tested to determine their plausible role in FD.

### 2.2. Identification of Fusarium kuroshium Metabolites

**Figure 2:**
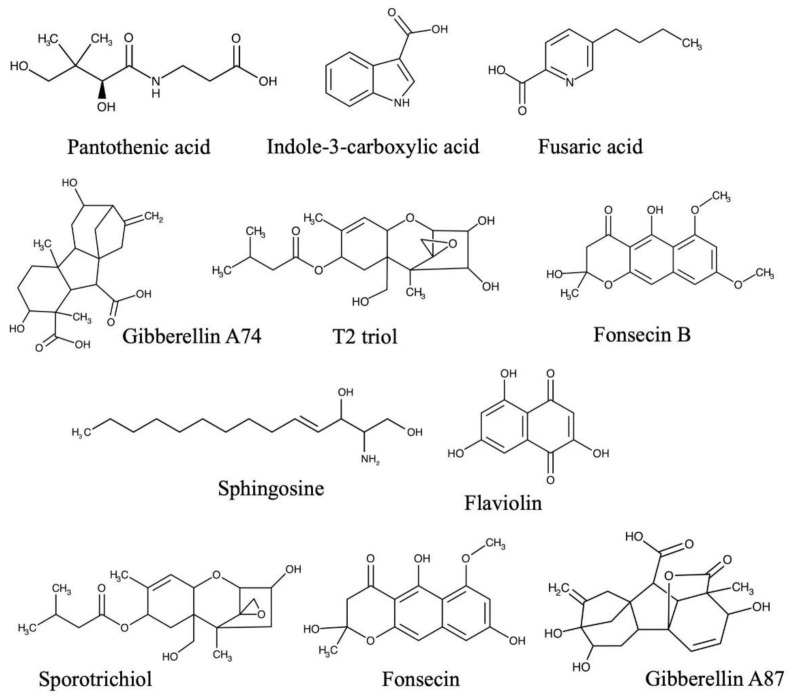
Chemical structures of fusaric acid (FA) and putatively identified compounds as constituents of the *F. kuroshium* exo-metabolome.

Molecules identified in the *F. kuroshium* exo-metabolome were grouped according to their possible roles in a plant–pathogen interaction and their chemical structures are presented in Figure 2. The first group is composed of the reported mycotoxins. For instance, FA (Supplementary materials, Figure S3), sporotrichiol (Supplementary materials, Figure S9) and T2-triol toxin (Supplementary materials, Figure S5) were identified in the EtOAc extract. Two plant growth regulators were also included in this group; Gibberellin A87 (Supplementary materials, Figure S11) and Gibberellin A74 (Supplementary materials, Figure S4). These regulators were identified in both *n*-BuOH and EtOAc extracts, respectively. The second group of metabolites identified in this study includes those compounds that are apparently unrelated to pathogenicity but with a plausible role in the plant–pathogen interaction, such as fonsecin (Supplementary materials, Figure S10) and fonsecin B (Supplementary materials, Figure S6), and were also identified in the EtOAc and *n*-BuOH extracts, respectively. In addition, the compound flaviolin (Supplementary materials, Figure S8) was identified in the EtOAc extract.

The following group comprised molecules with no known functions in a pathogenesis process but which contribute to the chemical knowledge of this *Fusarium* species. For example, pantothenic acid (Supplementary materials, Figure S1), indole-3-carboxylic acid (Supplementary materials, Figure S2) and sphingosine (Supplementary materials, Figure S7) were also identified in the EtOAc extract.

Finally, we analyzed the EtOAc and *n*-BuOH extracts through targeted metabolomics, employing calibration curves of reference standards of recognized toxins of the *Fusarium* genus, such as FA, fumonisins and trichothecenes. Using this approach, we identified and quantified FA in the EtOAc extract at 0.23 ± 0.01 µg L^−1^ (1.28 × 10^−6^ mM).

### 2.3. In Vitro Phytotoxicity Assay in Foliar Tissue of P. americana

**Figure 3:**
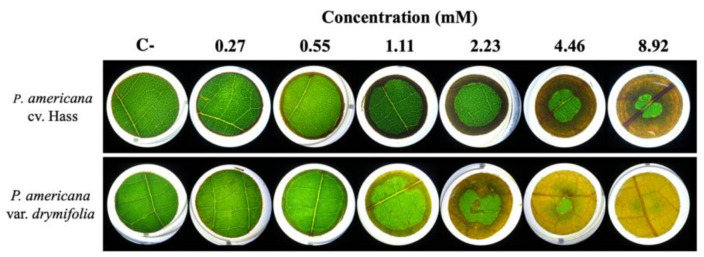
Phytotoxic effect of increasing concentrations of fusaric acid on leaves of *Persea americana* cv. Hass and var. *drymifolia* exposed for 96 h.

To determine whether organic extracts and FA provoke damage in avocado tissues, concentration-response experiments were performed on leaves of two avocado cultivars (*P. americana* var. *drymifolia* and *P. americana* cv. Hass) at different times (Supporting information Figure S12). First, in cv. Hass, FA did not cause any visible phytotoxic injury at low concentrations (<2.23 mM), but in var. *drymifolia*, chlorosis was observed from 1.11 mM of FA (Figure 3). This analysis allowed us to conclude that *P. americana* var. *drymifolia* is more susceptible to FA (*F* = 13.31; *p* < 0.001), while the generalized linear model (GLM) shows the foliar damage was influenced in both varieties by exposure time (*F* = 39.89, *p* < 0.001) and FA concentration (*F* = 297.89, *p* < 0.001).

### 2.4. Effect of F. kuroshium Extracts on P. americana Foliar Tissue

We evaluated whether the *F. kuroshium* extracts had a similar effect as the FA on both avocado varieties, using a commercially available FA (2 mM) standard as a positive control.

**Figure 4:**
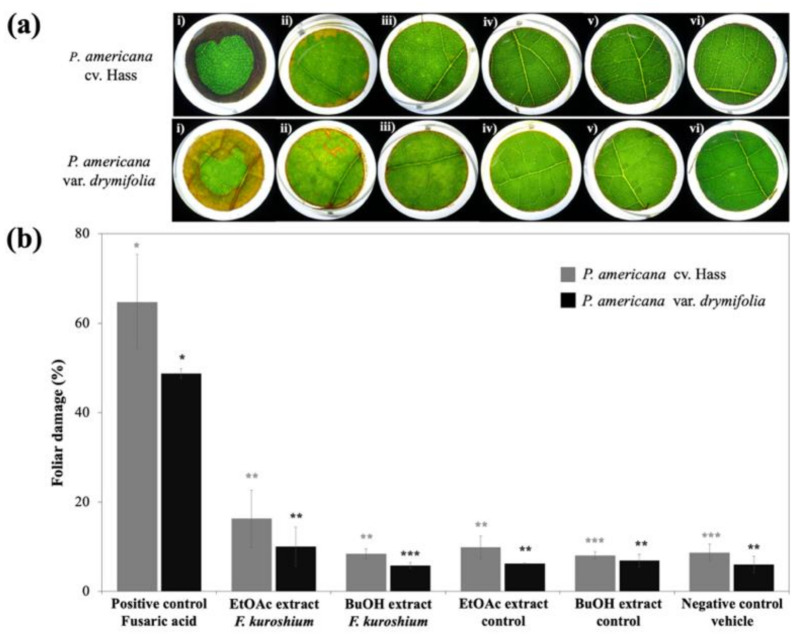
Phytotoxic effects (**a**) and foliar damage percentage (**b**) of *F. kuroshium* extracts tested on leaves of *P. americana*. (i) positive control 2 mM FA; (ii) EtOAc *F. kuroshium* extract; (iii) EtOAc control extract; (iv) *n*-BuOH *F. kuroshium* extract; (v) *n*-BuOH control extract; (vi) vehicle. Reported mean of three replicates (% ± SD). *, **, *** indicate statistically significant differences for each variety (*p* < 0.05).

Although no quantitative differences were detected between controls and *F. kuroshium* extracts (Figure 4b), and the effect was not as notorious as that displayed by the positive control (Figure 4ai; *F* = 7.71; *p* < 0.001), the EtOAc extract obtained from the cultured fungus caused slight damage to both varieties of avocado leaf discs (Figure 4aii). This suggests the presence of phytotoxic metabolites, at least in low concentration, and complements the description of the ten compounds described in this work for EtOAc extract. Finally, the *n*-BuOH extract did not cause any visual damage to either of the avocado varieties tested (Figure 4aiv), with this extract presenting only two identified compounds (fonsecin and gibberellin).

## 3. Discussion

### 3.1. Exo-Metabolome of F. kuroshium

In this study, we employed metabolomics tools to explore the metabolites responsible for the pathogenicity of this fungus. Analysis of the spectrometric features by PCA showed that in both ionization modes, the extracts displayed differences in their chemical composition. The volcano plots visually show the *F. kuroshium* chemical markers, and some putative identifications with different levels of accuracy were performed as a consequence.

The mycotoxins sporotrichiol and T2-triol have been previously reported in *Fusarium sporotrichioides* [23,27]. These belong to the trichothecenes group and are known as toxins that produce wilting, chlorosis and necrosis in plant hosts [30]. Gibberellin A87 and gibberellin A74 are diterpenoids belonging to a wide group of growth regulators in plants, many of them produced by several *Fusarium* species, such as *Fusarium fujikuroi* [22]. Some gibberellin-type molecules can act as virulence factors in plants, provoking alterations in normal plant growth and contributing to fungal colonization [31].

Other metabolites identified were fonsecin and fonsecin B, which belong to the naphtho-ɣ-pyrones. These compounds have been reported in the pathogenic fungi *Aspergillus* sp. and *Alternaria* sp. [32,33]. Fonsecin has antimicrobial activity against *Bacillus*, *Escherichia*, *Trichophyton* and *Candida* [34], while fonsecin B possesses free radical scavenging properties [24]. Fonsecin-related compounds have been isolated from *Fusarium* species, such as cetochromines, rubrofusarins, and ustilaginoidins [35]. The biosynthesis of these pigments by *F. kuroshium* could, therefore, be associated with defense mechanisms against competitive or antagonist microorganisms [34,36]. Moreover, the naphthoquinone flaviolin was also identified; its biosynthesis is related to rubrofusarin and norrubrofusarin, two compounds previously identified in *Fusarium* species. Flaviolin displays inhibitory activity against bacteria, yeasts, fungi and plant cells by interrupting cellular respiration and production of superoxide radicals [26].

Pantothenic acid is a vitamin that serves as a precursor for the biosynthesis of the CoA co-factor involved in fatty acids and carbohydrate metabolism and polyketide biosynthesis [19]. The indole-3-carboxylic acid was also identified. This is an aromatic compound reported in the endophytic fungus *Lasiodiplodia* sp. [20]. Interestingly, this molecule is a product of oxidative decarboxylation of indole-3-acetic acid [37], an auxin involved in plant growth, and was reported in *Fusarium graminearum* [38] and *Fusarium delphinoides* [39]. Finally, sphingosine, a compound involved in the biosynthesis of structural components, such as sphingolipids, was also annotated in *F. kuroshium* exo-metabolome [27].

### 3.2. Identification of FA

Through untargeted metabolomics, an *m/z* ratio of 180.1016 was identified, which may correspond to the [M+H]^+^ adduct of FA. The identity of FA was subsequently confirmed by targeted analysis focusing on the identification and quantification of 11 commercially available mycotoxins. This molecule is biosynthesized through the acetate-malonate pathway by the enzymatic activity of Fub 1, 3, 4 and 5 [40]. FA production capacity is widespread among several *Fusarium* species, such as *F. moniliforme*, *F. subglutinans*, *F. oxysporum*, *F. solani*, *F. proliferatum* and others [21,41]. The accurate identification of FA in *F. kuroshium* supports the results reported by Sánchez-Rangel and cols. [17], who identified the genes encoding the responsible enzymes of FA biosynthesis in this species. Identification of FA, added to the large number of *m/z* values obtained by the untargeted analysis, shows the possibility that *F. kuroshium* may synthesize novel molecules with unknown functions.

### 3.3. Phytotoxicity of FA and EtOAc Extract of F. kuroshium

The symptoms observed by FA's action resemble those previously reported in leaves of tomato plants and cucumber seedlings with FA at 1.4 mM and 0.27 mM, respectively [42,43]. This toxin has some phytotoxic properties reported in various plant species, including the capacity to provoke programmed cell death in tobacco [44], foliar wilting in tomato [45], and Liu and cols. [46] recently proved that FA acts as a virulence factor in bananas by decreasing O_2_ uptake, accumulating reactive oxygen species, increasing nuclear condensation and the loss of mitochondrial membrane potential, which acts to trigger cell death.

The effect displayed by the EtOAc extract raises the possibility to propose that *F. kuroshium* produces other toxin-type molecules with phytotoxic attributes, different from FA. This is also confirmed since the quantification of FA revealed that the compound is present in a lower amount (1.28 × 10^−6^ mM) and the in vitro assay showed that the lowest concentration to provoke necrosis in foliar tissue was 2 mM. In consequence, the presence of other unknown and novel phytotoxic components must not be discounted since *F. kuroshium* is an emerging and unknown plant pathogen.

## 4. Conclusions

*F. kuroshium*, the fungal partner in the ambrosia beetle complex, can produce several phytotoxic metabolites, such as FA and other mycotoxins, and these fungal organic extracts produce foliar damage under laboratory conditions in avocado leaves. Although the molecular mode of action of FA is unknown, its phytotoxic activity on several host plants is clear, and it remains to be demonstrated whether this toxin is produced in planta and if it is a critical player during FD establishment and course. Finally, the production of several unknown compounds in *F. kuroshium* extracts may reveal novel molecules with biological activities of interest. These findings and future studies will allow us to understand the role of *F. kuroshium* in the establishment and development of *Fusarium* dieback, as well as the molecular mechanism of the symbiosis. For example, it will be interesting to probe the effect of the different fungal molecules in the biology of ambrosia beetles. The approaches used in this study provide evidence of the role of *F. kuroshium* in FD disease and contribute to developing tools to mitigate the impact of this pathogen.

## 5. Materials and Methods

### 5.1. In Vitro Culture of Fusarium kuroshium and Its Exo-Metabolome Extraction

The Secretariat of Agriculture and Rural Development (SADER), through the National Service for Agro-Alimentary Public Health, Safety and Quality (SENASICA) and the General Directorate of Plant Health (DGSV) of the Mexican government provided the *F. kuroshium* strain HFEW-16-IV-019 and allowed us to perform the experiments at the National Center for Phytosanitary Reference (CNRF), under biosafety level 2 (BSL-2). The fungus was isolated from the mycangia of the KSHB beetle in Tijuana, Mexico and identified as *F. kuroshium* by [9]. The fungal isolate was grown on potato dextrose agar (PDA) medium for four days, before being inoculated for 10 days in 1.5 L of liquid rice media (2% *m/v* in deionized water) and incubated at 28 °C under constant agitation at 125 rpm (Thermo Scientific™ maxQ™ 4000, Waltham, MA, USA). The culture media was centrifuged (HERMLE Labortechnik Z200A, Wehingen, Germany) at 2800× *g* for 30 min, and the supernatant filtered through grade GF/B Whatman^®^ paper. Afterward, the supernatant was exhaustively extracted by liquid–liquid partitioning, first with EtOAc and then with *n*-butanol (*n*-BuOH). The organic phases in each partitioning separation were combined, passed through anhydrous Na_2_SO_4_ and concentrated by rotary evaporation under reduced pressure (BÜCHI RII, Flawil, Switzerland), yielding 63 mg and 98.5 mg from the EtOAc and *n*-BuOH extracts, respectively. The same procedure was applied to culture media without fungal inoculum, and this was used as a control in subsequent analysis.

### 5.2. Analysis of Exo-Metabolome by Untargeted and Targeted Metabolomics Based on Mass Spectrometry

Untargeted analysis of *F. kuroshium* exo-metabolome was performed with an ultra-high performance liquid chromatograph (UPLC; Waters™ Acquity Class I, Milford, MA, USA) coupled to a high-resolution mass spectrometer with a quadrupole-time of flight (QTOF) analyzer (Waters™ HDMi Synapt G2-Si, Milford, MA, USA), following the method reported by [47]. An electrospray ionization (ESI) source in positive and negative modes was employed. Intensity and retention time (Rt) for each mass-charge ratio (*m/z*) were acquired and processed with the software MassLynx™ version 4.1 (Milford, MA, USA). Principal component analysis (PCA), -fold change and *t*-test were performed with MetaboAnalyst 4.0 (Ste. Anne de Bellevue, QC, Canada) bioinformatics platform [48]. For putative identifications, the *m/z* values of *F. kuroshium* chemical markers, obtained by comparison with the uninoculated medium, were sought in public databases (Metlin [49], MassBank [50] and FooDB [51]) and the mass spectra were compared with the theoretical and experimental information for each compound with a maximum mass error allowed of 5 ppm.

In addition, targeted metabolomics analyses were performed on a UPLC (Agilent Technologies 1290 Infinity series, Santa Clara, CA, USA) coupled to a mass spectrometer with a triple quadrupole (QqQ) analyzer (Agilent Technologies 6460, Santa Clara, CA, USA). Calibration curves with authentic standards were generated to identify 11 mycotoxins (fusaric acid, beauvericin, deoxynivalenol, enniatins A, B and B1, fumonisins B1 and B2, moniliformin, verrucarin A and zearalenone). The acquisition method for the MS/MS fingerprints of reference compounds was dynamic multiple reaction monitoring (dMRM). Chromatographic separation was achieved using a reversed-phase column (Agilent Zorbax SB-C18; 1.8 µm, 2.1 × 50 mm) and the mobile phase (flow rate of 0.3 mL min^−1^) consisted of a mixture of two solutions of 0.1% (*v*/*v*) formic acid, one in water (A) and the other in acetonitrile (B). The gradient started with 10% of B and was maintained for 3 min before continuously increasing to 70% of B from 3 to 10 min. Afterward, B was increased to 90% from 10.1 to 12 min. Finally, B was decreased to 10% from 12.1 to 15 min. One µL of extract of each sample was injected in triplicate at a final concentration of 20 mg mL^−1^ dissolved in 0.1% (*v*/*v*) of formic acid in methanol (both MS grade). The spectrometric analyses were conducted by electrospray ionization (ESI) in positive and negative modes. For this, the capillary and injector voltages were 3.5 and 0.5 kV, respectively. The desolvation nebulizer had a pressure of 45 psi, the sheath gas (N_2_) was used at 350 °C with a flow of 11 L min^−1^, the fragmentor voltage was 100 V, and the collision energy was optimized individually for each compound. The concentration range tested was from 0.25 to 17 μM, and each point was injected in triplicate. Values of R^2^ > 0.99 were obtained. Transition values of precursor and product ions are described in Supplementary Materials, Table S1, and were selected according to the values reported in public databases. Quantification was performed with the software MassHunter Workstation version B.06.00 (Agilent Technologies).

### 5.3. In Vitro Assay for Determination of Fungal Extracts and Fusaric Acid Phytotoxicity

Avocado (*Persea americana* Mill.) leaves showing no damage were retrieved from one-year-old plants, cv. Hass and var. *drymifolia*. Avocado plants were obtained from a commercial greenhouse located in Uruapan in Michoacán, Mexico, and kept in semi-controlled conditions in an experimental greenhouse at 25 °C and 68% relative humidity (RH) in Cluster BioMimic^®^, INECOL. The phytotoxicity assay was performed as reported by Desjardins [52], with modifications. Leaves of each plant were surface disinfected in a 2% (*v*/*v*) solution of commercial bleach (NaClO) in sterile distilled water (dH_2_O), and circles of 15 mm diameter were then cut from the leaves and placed in 12-well plates containing 1 mL of 0.09% (*w*/*v*) of Murashige and Skoog medium (Phytotechnology Laboratories, Cat. M524) enriched with 1.5% (*w*/*v*) of sucrose in dH_2_O, pH = 7.00, plus the different treatments.

For evaluating FA phytotoxicity, concentration-response experiments were developed at 0.1, 0.2, 0.5, 1.1, 2.2, 4.4 and 8.9 mM using an authentic standard (Sigma-Aldrich, Cat. F6513). In addition, *F. kuroshium* organic extracts were evaluated at 1.5 mg mL^−1^ using FA in a final concentration of 2 mM as positive control and 50 μL of vehicle (EtOAc–DMSO–water (5:5:90)) as a negative control, which corresponds to the maximum volume used to deposit the extracts on avocado leaves. Each treatment was evaluated in triplicate. Plates were incubated in a growth chamber (Percival Scientific TE-36VL, Iowa, USA) at 22 °C, 65% RH and a 16:8 h light:dark photoperiod. Observations were performed using a stereoscopic microscope at 10×/20 (LEICA EZ4, Wetzlar, Germany) daily for four days to determine the progress of foliar damage, which was quantified using ImageJ2 software [53]. The results are reported as the average percentage of foliar damage ± standard deviation (% ± DE).

The data were ranked and analyzed in R-studio software [54] by two-way ANOVA for determination of the phytotoxicity of organic extracts in tested plants and, in the case of FA, it was used a generalized linear model (GLM) to identify the factor (concentration, variety and time) with the most influence on the damage of this mycotoxin.

## Figures and Tables

**Table 1:** Compounds putatively identified in *F. kuroshium* exo-metabolome.

RT (min)	Extract	Molecular Formula of *m/z* (Δ ppm)	Parent Ion Mass *m/z*	MS/MS	Level ID	Putative ID/Chemical Family	Reference
1.73	EtOAc	C_9_H_17_NO_5_ (1.4)	220.1181 [M+H]^+^	202.1080, 184.0968, 172.0970	2	Pantothenic acid/ Secondary alcohols	[19]
1.75	EtOAc	C_9_H_7_NO_2_ (−0.6)	162.0551 [M+H]^+^	144.0444, 90.0527	2	Indole-3-carboxylic acid/ Indole carboxylic acids	[20]
3.35	EtOAc	C_10_H_13_NO_2_ (1.6)	180.1016 [M+H]^+^	-	3	Fusaric acid/ Picolinic acid	[21]
4.74	EtOAc	C_20_H_28_O_6_ (−2.7)	365.1955 [M+H]^+^	347.1853, 319.1903, 303.1954	2	Gibberellin A74/ Diterpenoids	[22]
4.76	EtOAc	C_20_H_30_O_7_ (3.4)	381.1912 [M−H]^−^	351.1799, 193.0498	2	T2 triol/ Trichothecenes	[23]
5.22	EtOAc	C_16_H_16_O_6_ (3.9)	303.0868 [M−H]^−^	-	3	Fonsecin B/ Naphtho-gamma-pyrones	[24]
5.59	EtOAc	C_14_H_30_NO_2_ (−0.4)	244.2273 [M+H]^+^	-	3	Sphingosine/ Amino alcohols	[25]
5.69	EtOAc	C_10_H_6_O_5_ (3.9)	205.0133 [M−H]^−^	177.0184, 175.0389, 165.0549, 151.0388	2	Flaviolin/ Naphthoquinones	[26]
6.33	EtOAc	C_20_H_30_O_6_ (−3.60)	389.1921 [M+Na]^+^	-	3	Sporotrichiol/ Trichothecenes	[27]
6.35		C_20_H_30_O_6_ (4.65)	365.1970 [M−H]^−^	-			
7.11	EtOAc and *n*-BuOH	C_15_H_14_O_6_ (3.8)	291.0858 [M+H]^+^	273.0752, 249.0753, 231.0647,189.0540	2	Fonsecin/ Naphtho-gamma-pyrones	[28]
9.88	*n*-BuOH	C_19_H_22_O_7_ (1.0)	385.1252 [M+Na]^+^	-	3	Gibberellin A87/ Diterpenoids	[22]

The MS/MS data were compared with the fragmentation modeling for metabolite identification [29]. Δ ppm = mass error.

## Data Availability

All data are contained within this article and Supplementary Materials.

## Supplementary Materials

The following are available online at https://www.mdpi.com/article/10.3390/toxins13040268/s1, Figure S1: HR-ESI-MS of pantothenic acid, Figure S2: HR-ESI-MS of indole-3-carboxylic acid, Figure S3: HR-ESI-MS of fusaric acid, Figure S4: HR-ESI-MS of gibberellin A74, Figure S5: HR-ESI-MS of T2 triol toxin, Figure S6: HR-ESI-MS of fonsecin B, Figure S7: HR-ESI-MS of sphingosine, Figure S8: HR-ESI-MS of flaviolin, Figure S9: HR-ESI-MS of sporotrichiol, Figure S10: HR-ESI-MS of fonsecin, Figure S11 HR-ESI-MS of gibberellin A87, Figure S12: Concentration-response curves of FA on foliar tissue of *P. americana* cv. Hass (a) and var. *drymifolia* (b). Table S1: Optimized dMRM parameters for detection of mycotoxins. File S1: Mass features secreted by *F. kuroshium*, detected by HR-ESI-MS.
