# Supplementary material for: Characterization of the Exo-Metabolome of the Emergent Phytopathogen Fusarium kuroshium sp. nov., a Causal Agent of Fusarium Dieback
Source: Toxins (Basel). 2021 Apr 9;13(4):268. doi: 10.3390/toxins13040268 (PMC8069249; doi:10.3390/toxins13040268)
Supplement: Supplementary file 1 [file toxins-13-00268-s001.zip › toxins-1138828 SU layout .docx]

Supplementary Materials: Identification and Characterization of Potential Virulence Factors of the Emergent Phytopathogen *Fusarium kuroshium* sp. nov., a Causal Agent of Fusarium Dieback

Angélica Gutiérrez-Sánchez, Javier Plasencia, Juan L. Monribot-Villanueva, José B. Rodríguez-Haas, Jose Abel López-Buenfil, Clemente J. García-Ávila, Eliel Ruiz-May, Diana Sánchez-Rangel and José A. Guerrero-Analco


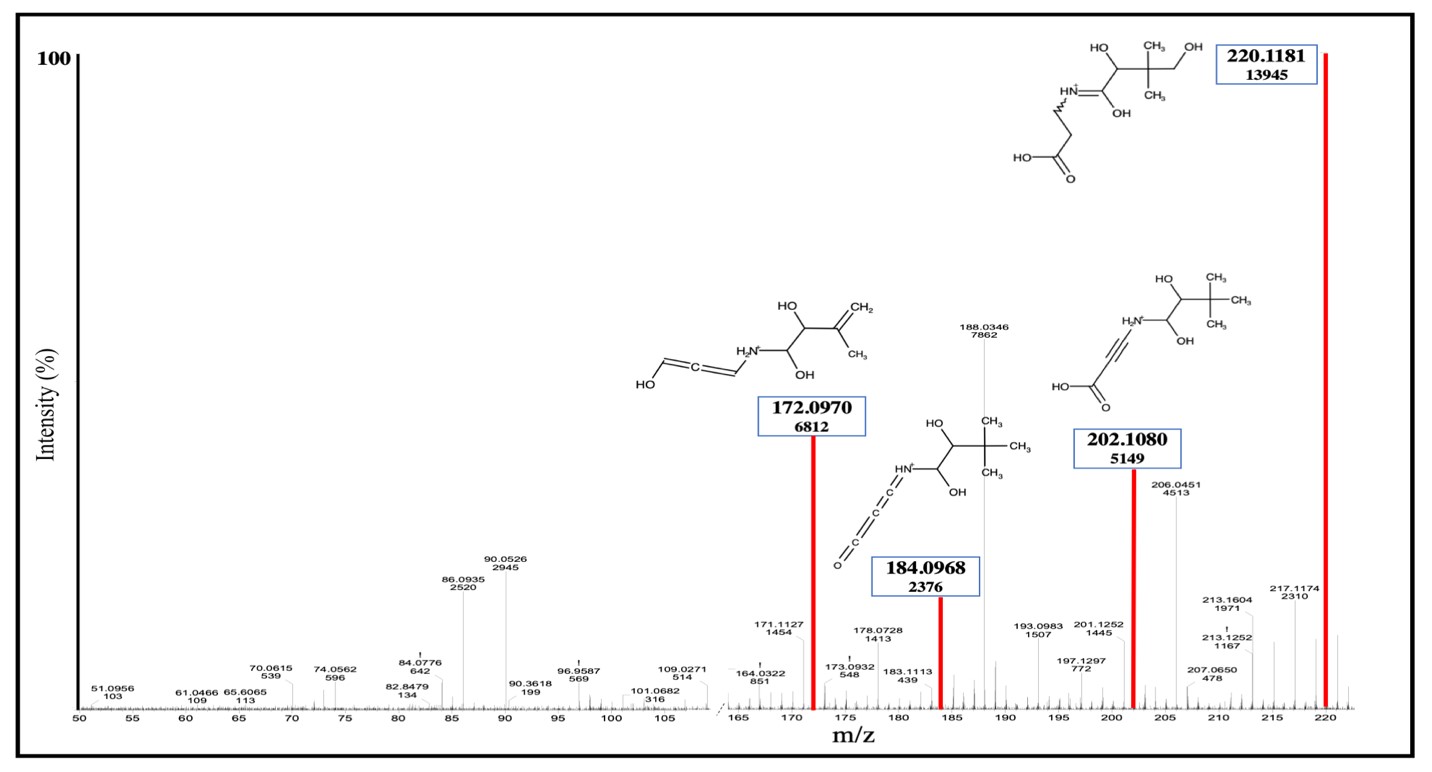


**Figure S1.** HR-ESI-MS of pantothenic acid.


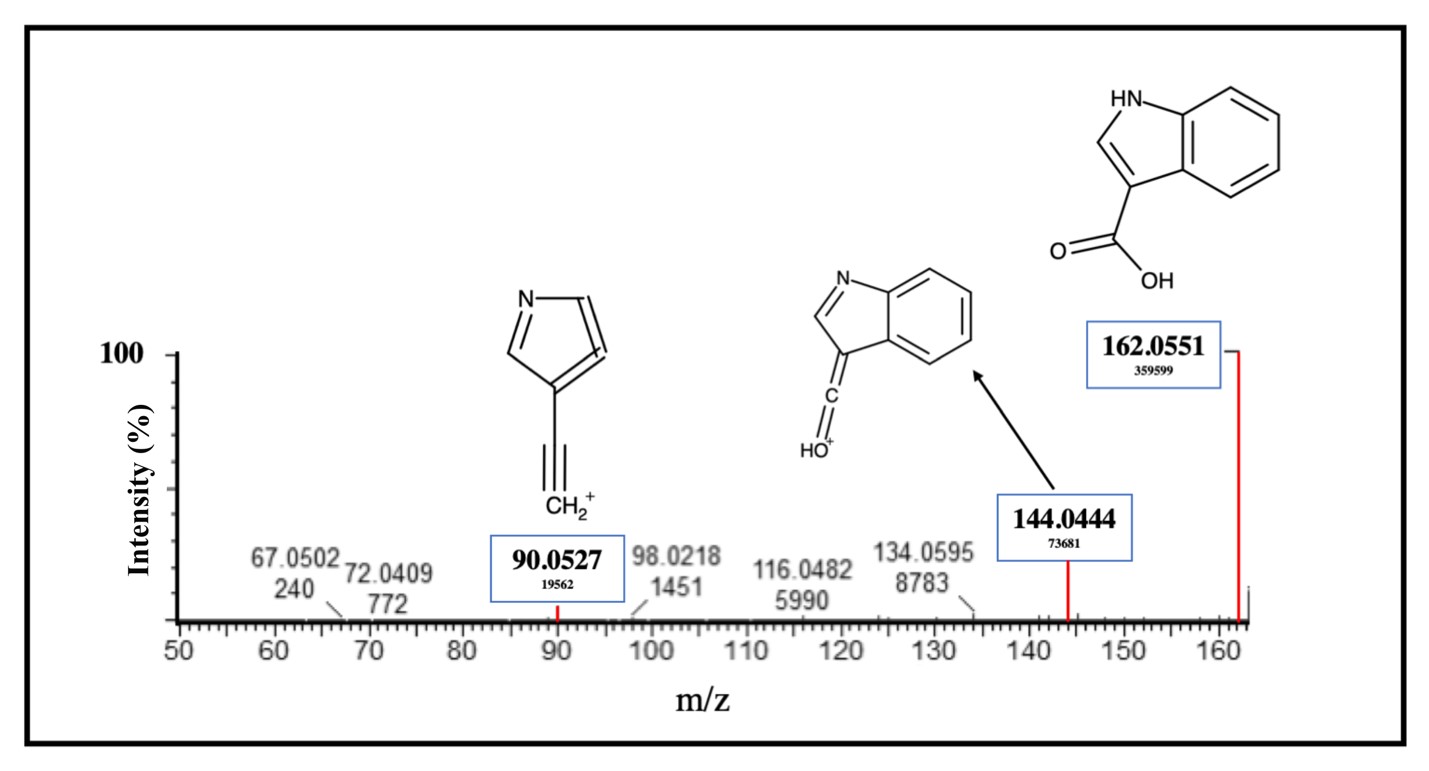


**Figure S2.** HR-ESI-MS of indole-3-carboxylic acid.


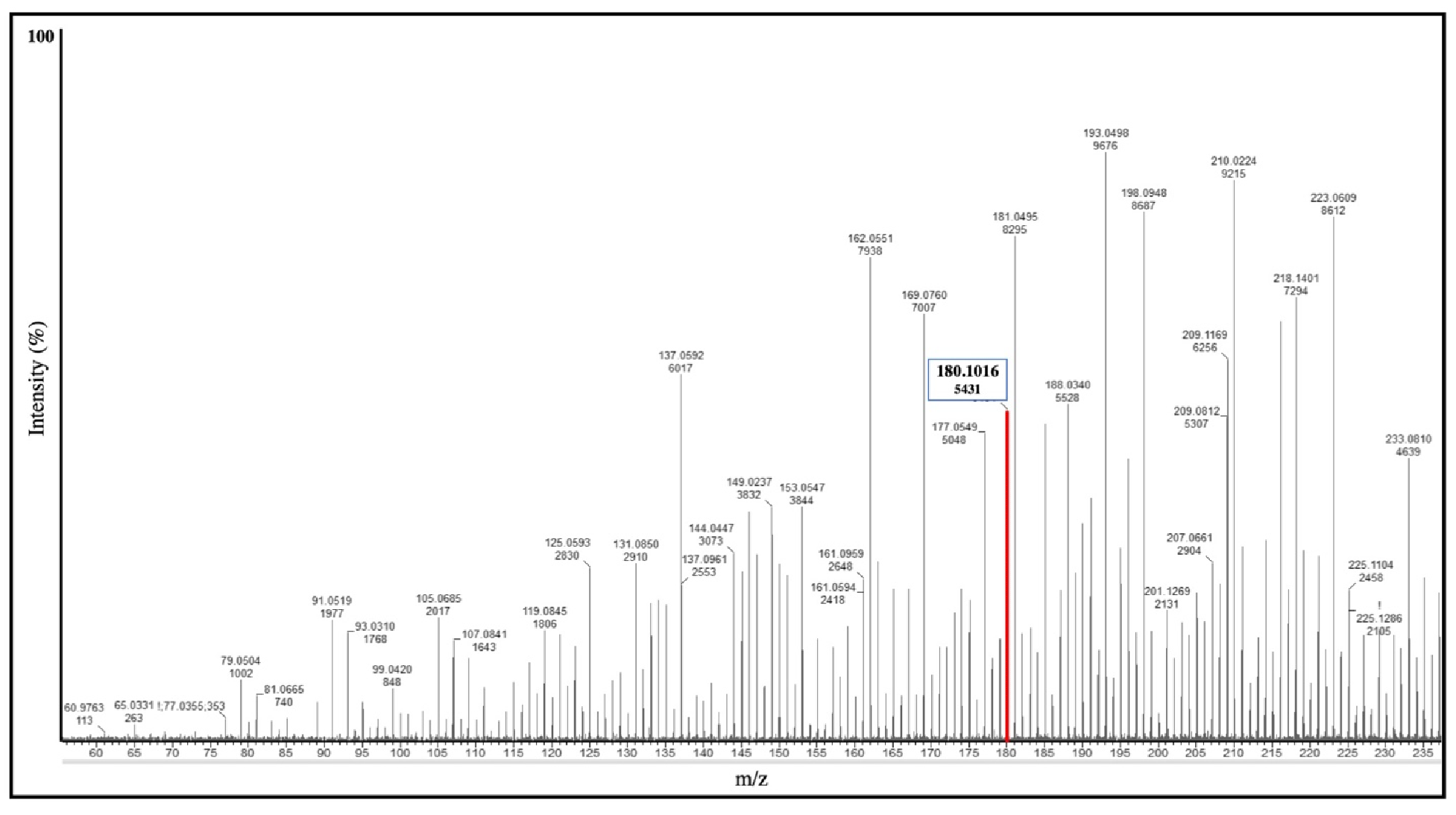


**Figure S3.** HR-ESI-MS of fusaric acid.


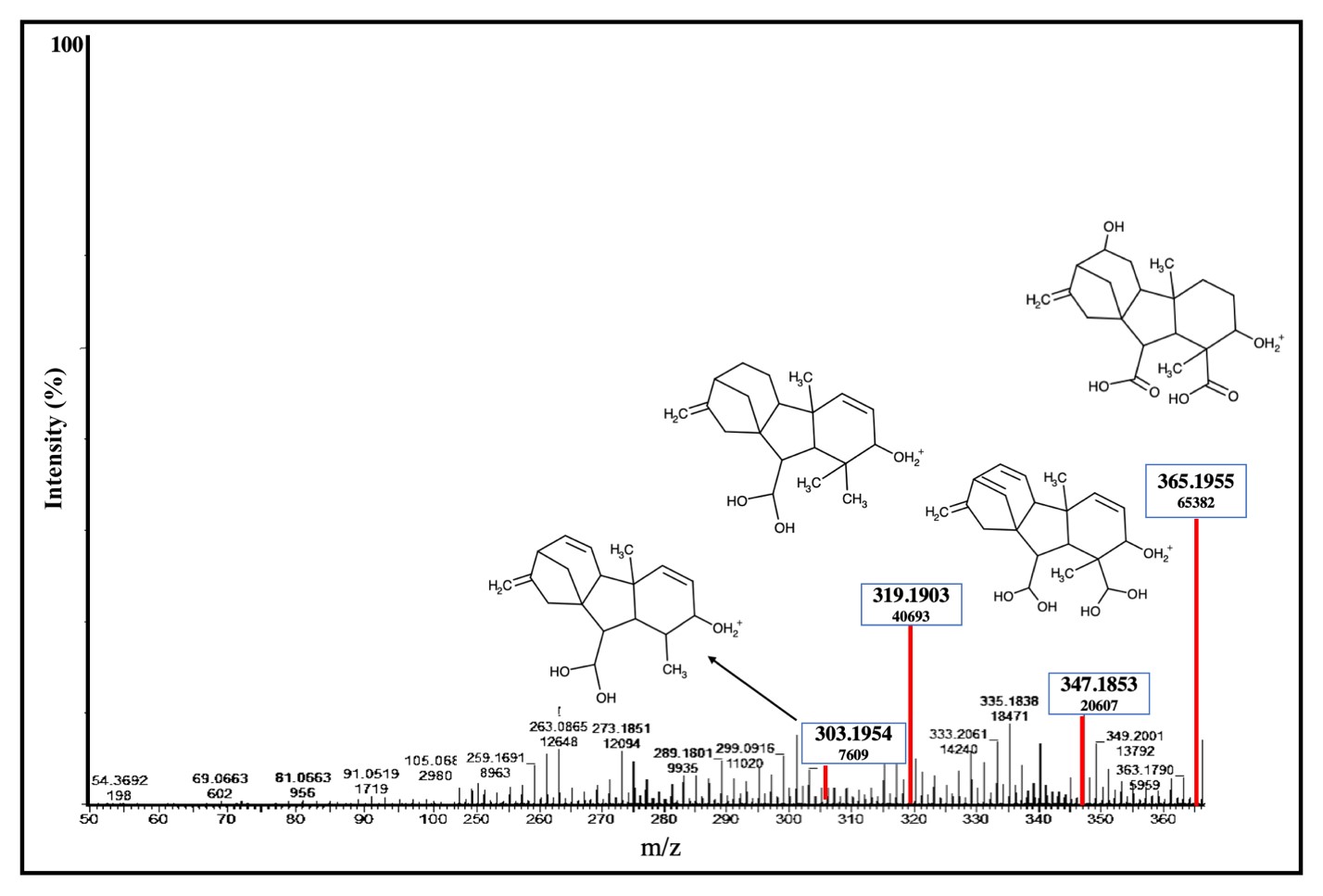


**Figure S4.** HR-ESI-MS of gibberellin A74.


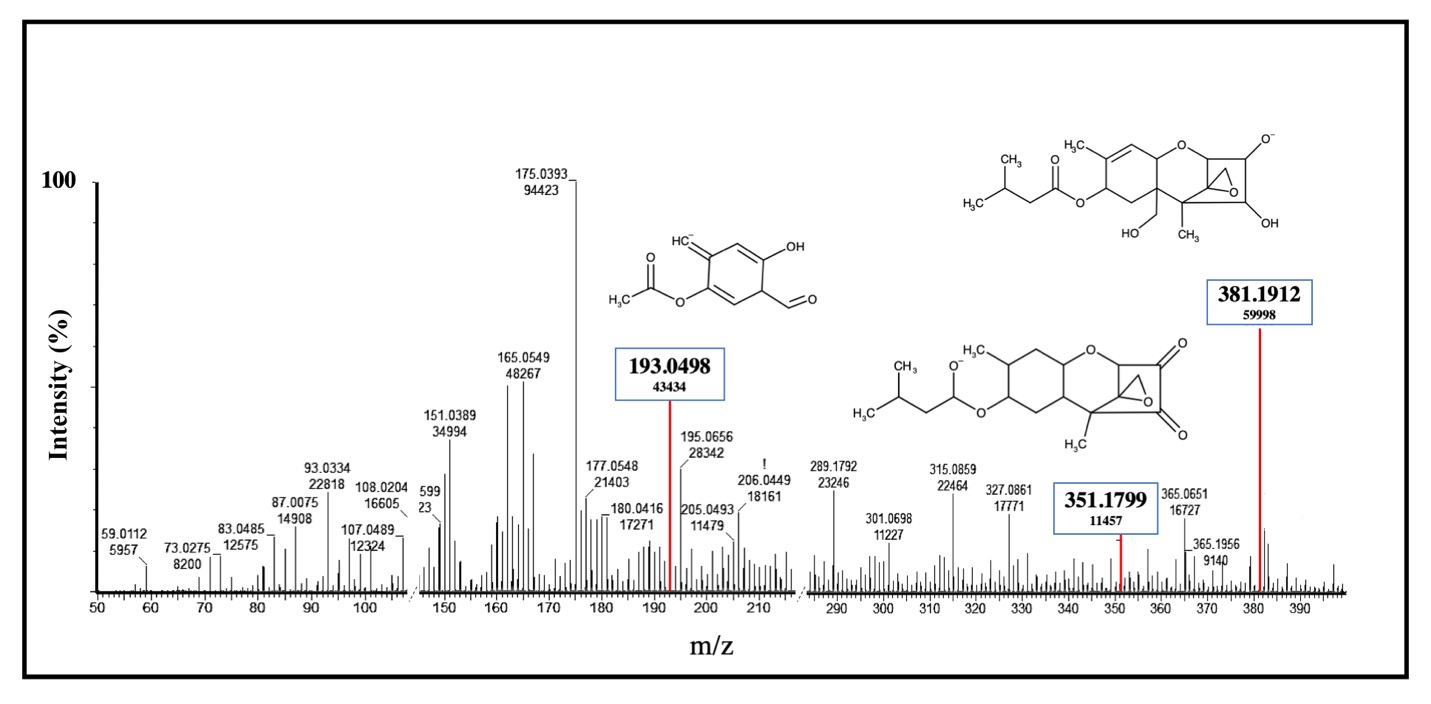


**Figure S5.** HR-ESI-MS of T2 triol.


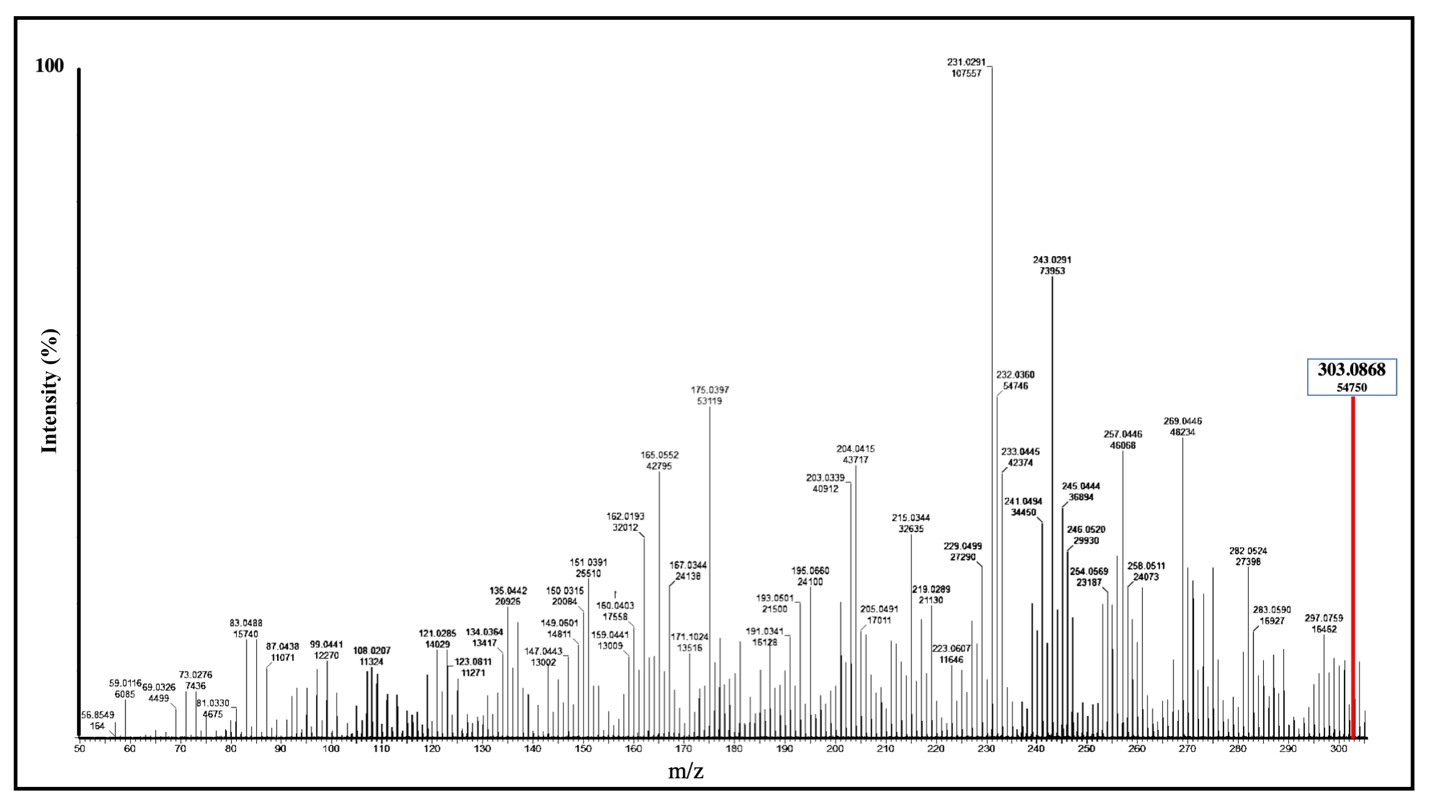


**Figure S6.** HR-ESI-MS of fonsecin B.


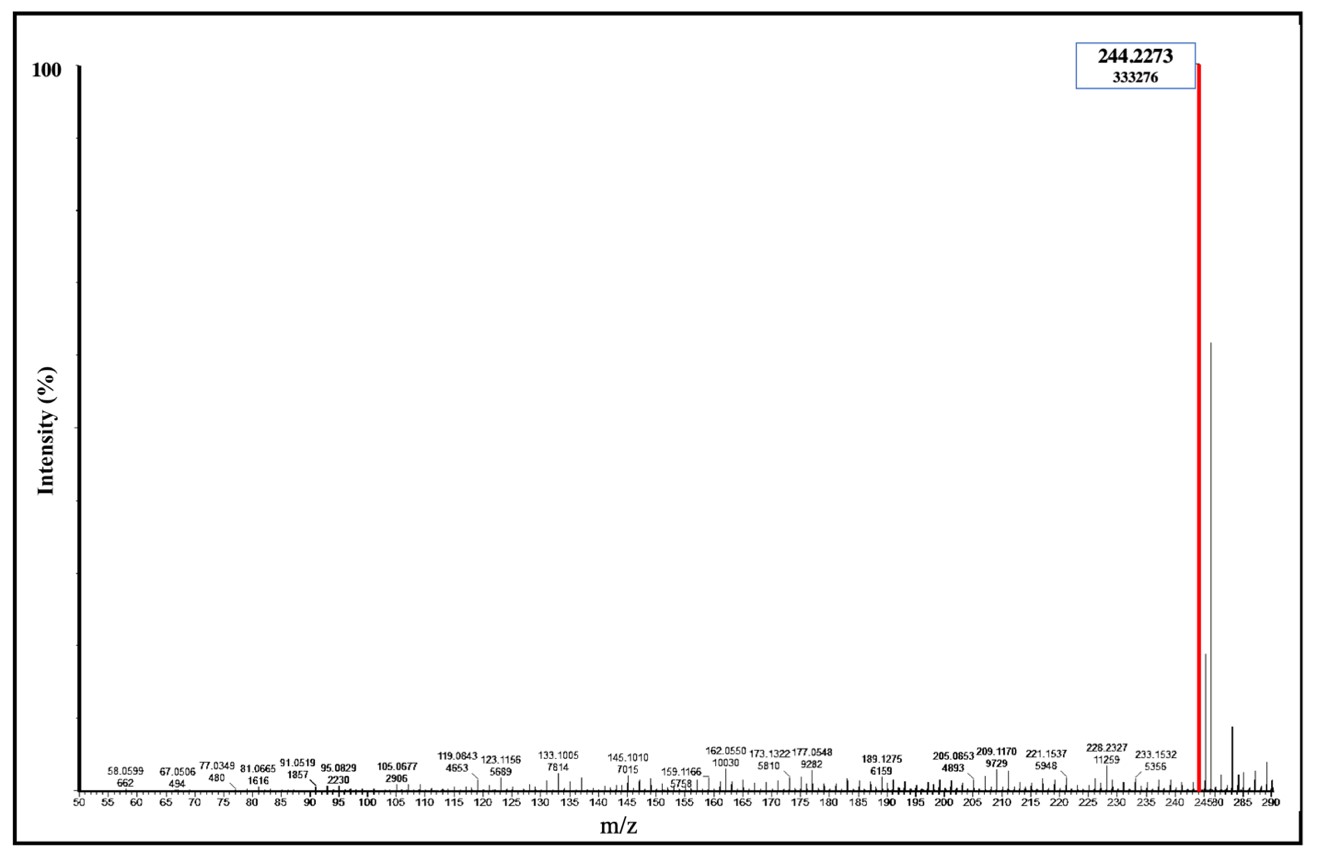


**Figure S7.** HR-ESI-MS of sphingosine.


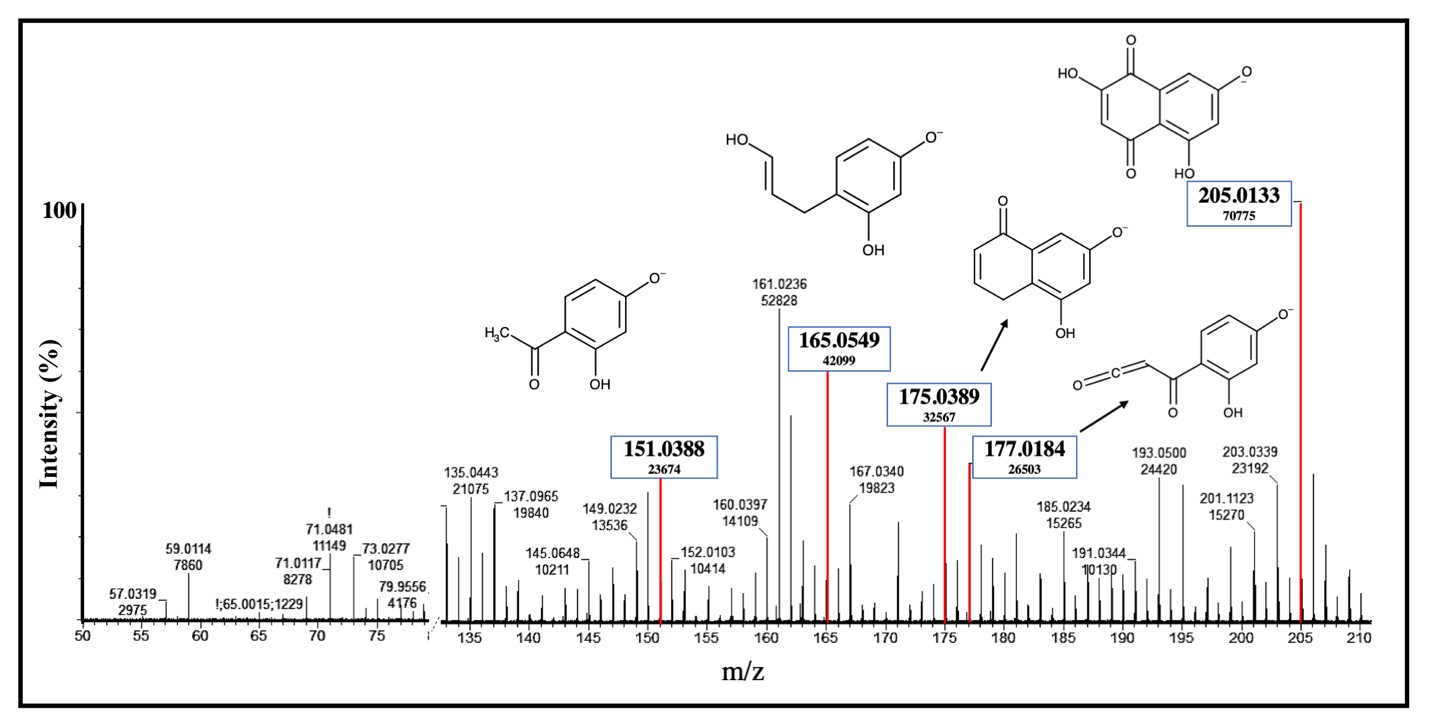


**Figure S8.** HR-ESI-MS of flaviolin.


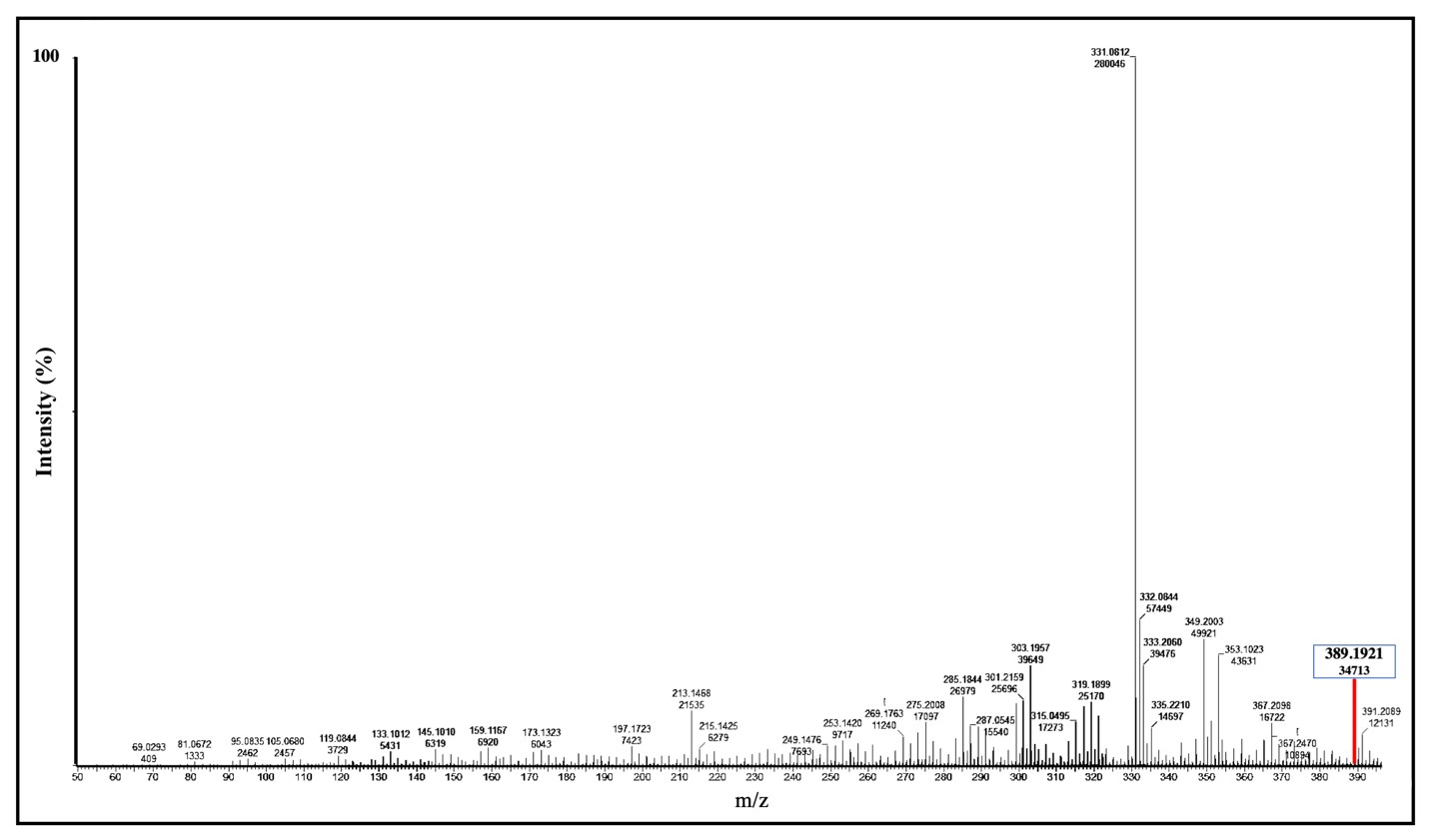


**Figure S9.** HR-ESI-MS of sporotrichiol.


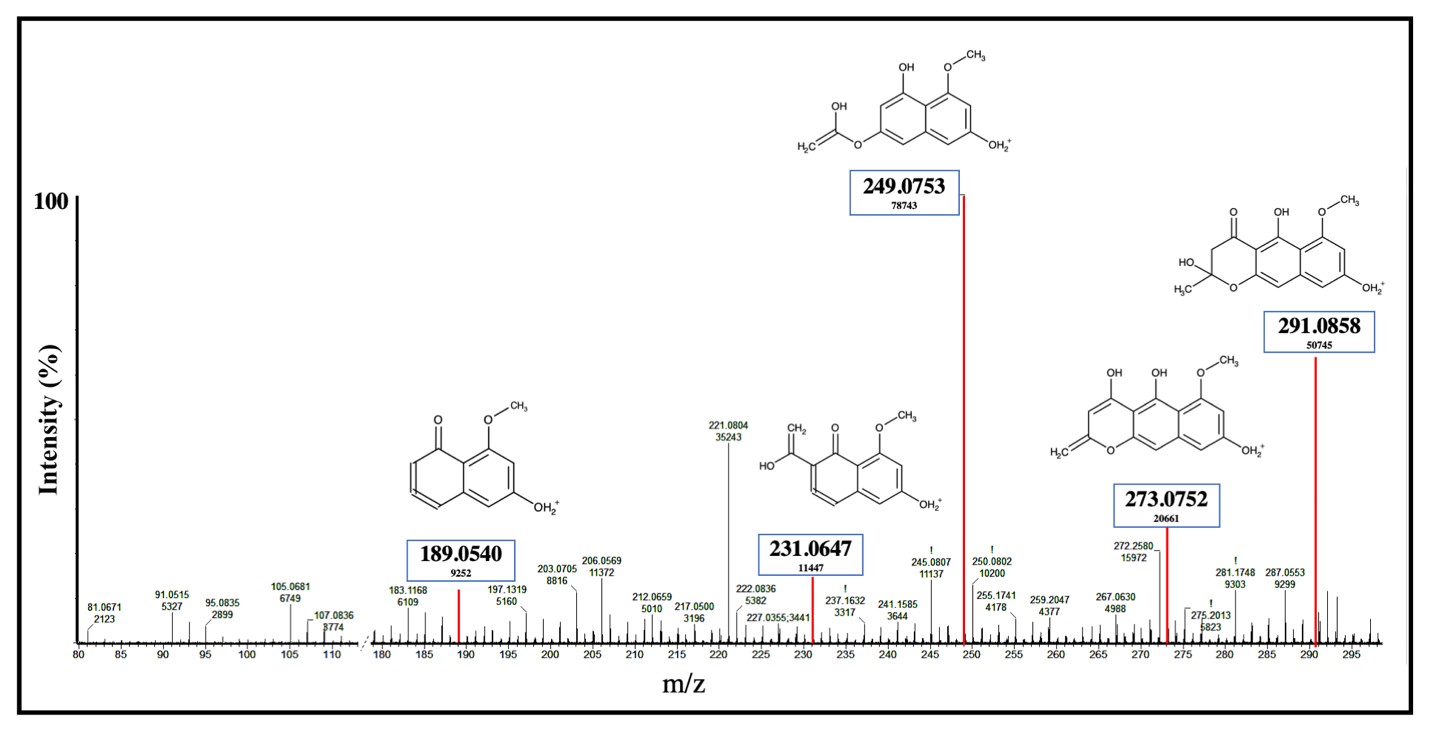


**Figure S10.** HR-ESI-MS of fonsecin.


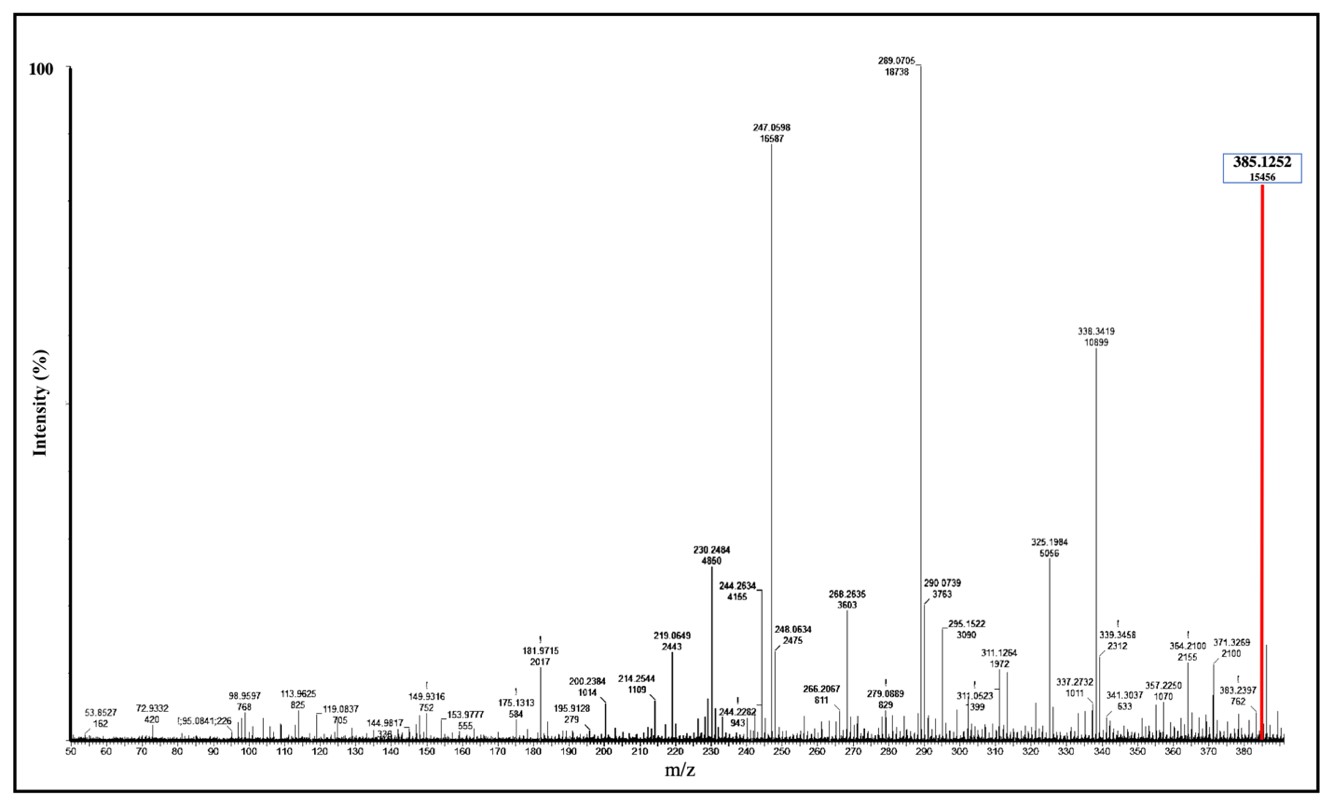


**Figure S11.** HR-ESI-MS of gibberellin A87 .


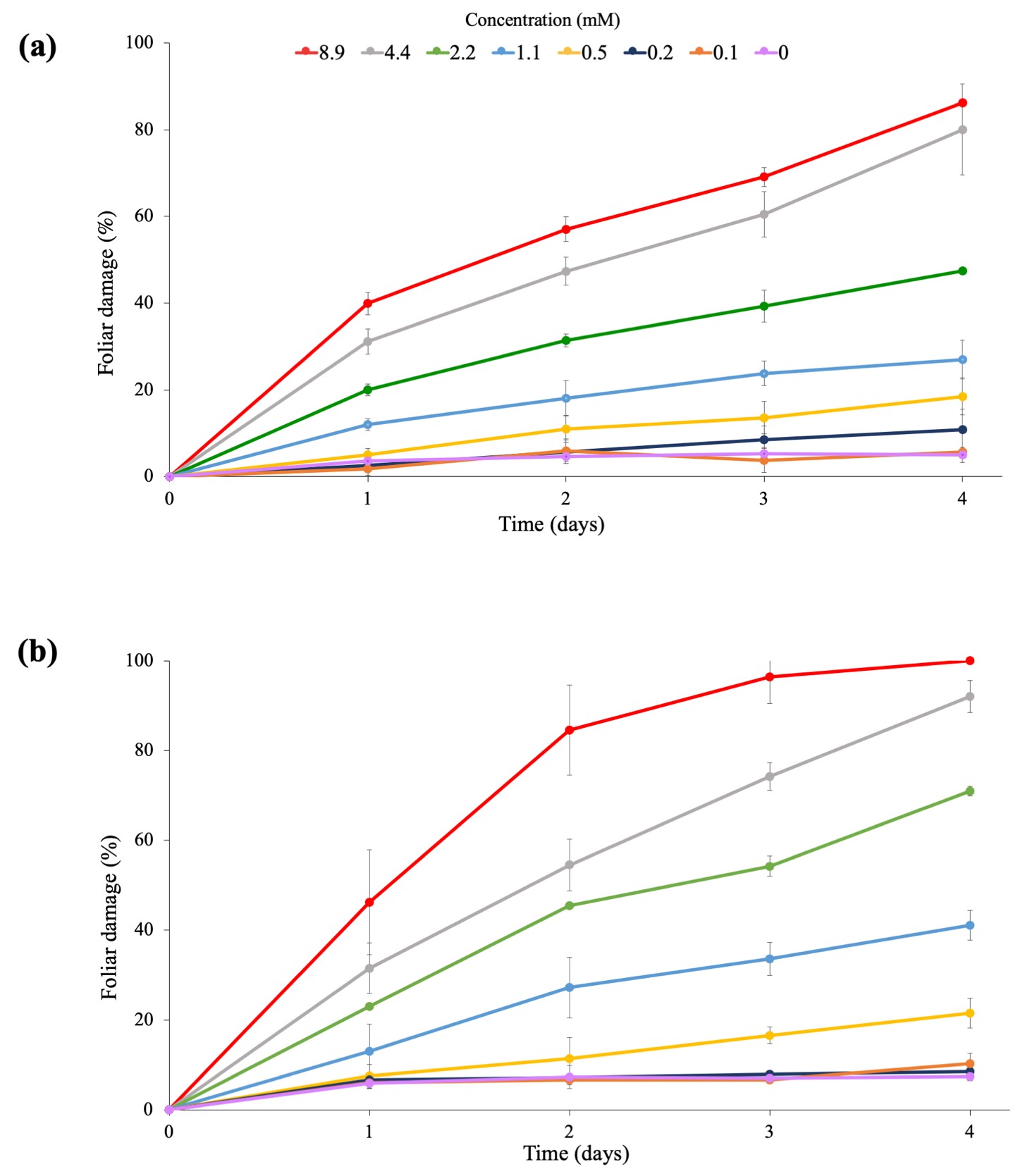


**Figure S12.** Concentration-response curves of FA on foliar tissue of *P. americana* cv. Hass (**a**) and var. *drymifolia* (**b**).

**Table S1.** Optimized dMRM parameters for detection of mycotoxins.

| **Mycotoxin** | **Parent Ion (*m/z*)** | **Product Ion (*m/z*)** | **Polarity** | **Collision Voltage (V)** | **Retention Time (min)** | **Concentration Range (µM)** | **R^2^** |
| --- | --- | --- | --- | --- | --- | --- | --- |
| Fusaric acid | 180.10 | 134.09 | + | 20 | 5.80 | 0.25–17 | 0.996 |
| Beauvericin | 784.40 | 244.10 | + | 40 | 11.84 | 0.5–17 | 0.996 |
| Deoxynivalenol | 297.20 | 231.20 | + | 05 | 4.82 | 0.5–17 | 0.996 |
| Enniatin A | 682.60 | 228.26 | + | 40 | 12.50 | 0.5–17 | 0.996 |
| Enniatin B | 640.40 | 196.20 | + | 30 | 11.42 | 0.5–17 | 0.995 |
| Enniatin B1 | 654.40 | 214.20 | + | 35 | 11.71 | 0.5–17 | 0.996 |
| Fumonisin B1 | 722.40 | 352.30 | + | 40 | 7.33 | 0.5–17 | 0.996 |
| Fumonisin B2 | 706.40 | 336.30 | + | 40 | 8.01 | 0.5–17 | 0.996 |
| Moniliformin | 96.90 | 41.20 | - | 24 | 0.68 | 0.25–17 | 0.996 |
| Verrucarin A | 503.20 | 249.00 | + | 10 | 8.60 | 0.5–17 | 0.996 |
| Zearalenone | 319.20 | 301.10 | + | 10 | 8.90 | 1–17 | 0.996 |
